# Energetic Aspects and Molecular Mechanism of 3-Nitro-substituted 2-Isoxazolines Formation via Nitrile N-Oxide [3+2] Cycloaddition: An MEDT Computational Study

**DOI:** 10.3390/molecules29133042

**Published:** 2024-06-26

**Authors:** Ewa Dresler, Aneta Wróblewska, Radomir Jasiński

**Affiliations:** 1 Łukasiewicz Research Network—Institute of Heavy Organic Synthesis “Blachownia”, Energetyków 9, 47-225 Kędzierzyn-Koźle, Poland; ewa.dresler@icso.lukasiewicz.gov.pl; 2 Department of Organic Chemistry, University of Lodz, Tamka 12, 91-403 Łódź, Poland; aneta.wroblewska@chemia.uni.lodz.pl; 3 Institute of Organic Chemistry and Technology, Cracow University of Technology, Warszawska 24, 31-155 Cracow, Poland

**Keywords:** [3+2] cycloaddition, isoxazolines, Gibbs free energy of activation, Gibbs free energy of reaction

## Abstract

Regioselectivity and the molecular mechanism of the [3+2] cycloaddition reaction between nitro-substituted formonitrile N-oxide **1** and electron-rich alkenes were explored on the basis of the wb97xd/6-311+G(d) (PCM) quantum chemical calculations. It was established that the thermodynamic factors allow for the formation of stable cycloadducts along all considered models. The analysis of the kinetic parameters of the main processes show that all [3+2] cycloadditions should be realized with full regioselectivity. In all cases, the formation of 5-substituted 3-nitro-2-isoxazolidines is clearly preferred. It is interesting that regiodirection is not determined by the local electrophile/nucleophile interactions but by steric effects. From a mechanistic point of view, all considered reactions should be treated as polar, one-step reactions. All attempts to locate the hypothetical zwitterionic intermediates along the cycloaddition paths were, however, not successful.

## 1. Introduction

Isoxazole ring, as well as its hydrogenated analogs (isoxazolines and isoxazolidines), are key molecular segments of many important bioactive structures such as *afoxolaner* [1], *lotilaner* [2], *sarolaner* [3], *fenoxasulfone* [4], and many others [5,6,7,8,9]. The most universal strategy for the preparation of five-membered heterocycles (including isoxazoles) is [3+2] cycloaddition (32CA) [10,11,12]. For the preparation of 2-isoxazolines, 32CA processes involving nitrile N-oxides as the Three-Atom Components (TACs) are used [13,14]. Within this group, nitro-functionalized structures play a special role because the presence of the nitro group opens a wide range of potential further functionalization spectrum [15,16,17,18,19]. The nitro group additionally stimulates many bioactive functions [18,20,21,22].

**Scheme 1:**
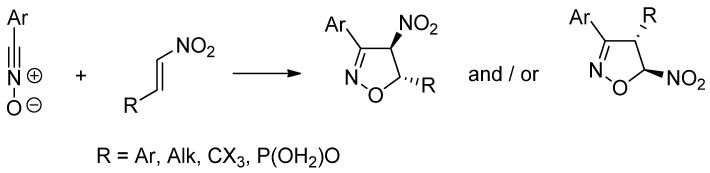
General regioselectivity in the 32CAs involving aromatic nitrile N-oxides to 2-substituted nitroethenes.

An effective way for the preparation of 4-nitro- and 5-nitro-2-isoxazoline molecular segments is the [3+2] cycloaddition reactions involving nitrile N-oxides and conjugated nitroalkenes [23,24,25,26]. Necessary nitroalkenes can be easily obtained from respective nitroalkanes and carbonyl compounds via the Henry reaction and further elimination of water molecules [27,28]. On the other hand, alkyl—or aryl nitrile N-oxides—can be prepared from commercially available aldehydes via the oxime formation and further halogenation/dehydrohalogenation sequence [29,30,31,32]. Consequently, a large number of effective protocols for the synthesis of 4 and 5-nitroisoxazolines is available in the current literature (Scheme 1).

**Scheme 2:**
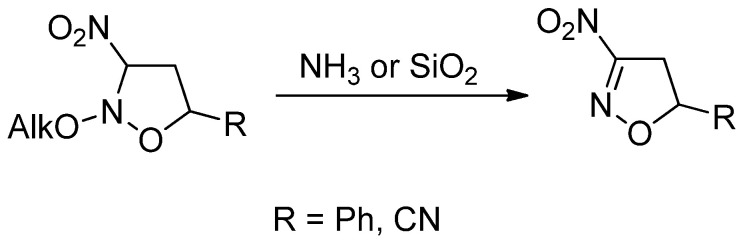
Oxidative conversion of 2-alkoxy-3-nitroisoxazolidines into 3-nitroisoxazoline derivatives.

The preparation of 3-nitro-2-isoxazolines is more difficult. At present, only some works discuss this important preparative problem. It is possible to eliminate hydroxyalkanes catalyzed by ammonia or silica dioxide [33,34]. Unfortunately, this approach requires the use of starting materials, which must be prepared using nitrosubstituted acyclic nitronates; thus, the preparation strategy is difficult [35] (Scheme 2).

**Scheme 3:**
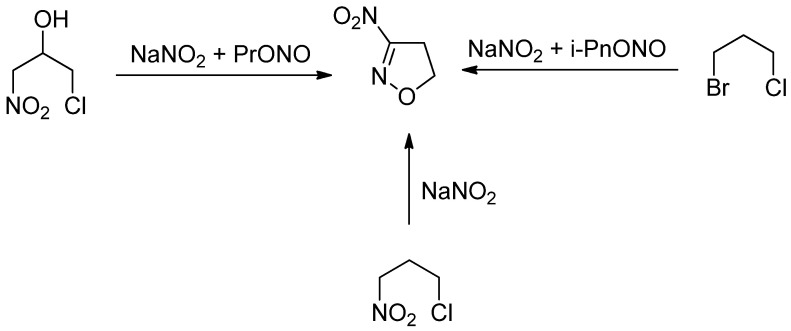
Conversion of 1,3-dihalopropane, 1-chloro-3-nitropropane, and 1-chloro-3-nitropropan-2-ol into 3-nitroisoxazoline derivatives.

The parent 3-nitro-2-isoaxzoline can alternatively be prepared via the cyclisation of 3-nitro-2-hydroxy-1-chloropropane or 3-bromo-1-chloropropane in the presence of sodium nitrite and alkyl nitrites [36,37] or via the cyclisation of 3-nitro-1-chloropropane in the presence of sodium nitrite [38] (Scheme 3).

**Scheme 4:**
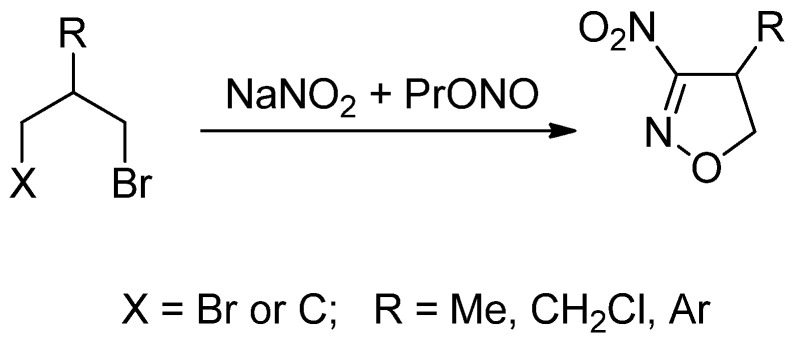
Conversion of 1,3-dihalo-3-R-propanes into 3-nitroisoxazoline derivatives.

Similarly, some 3-nitro-4-R-2-isoxazolines can be synthesized based on respective 1,2-dihalo-2-R-propanes [39] (Scheme 4).

**Scheme 5:**
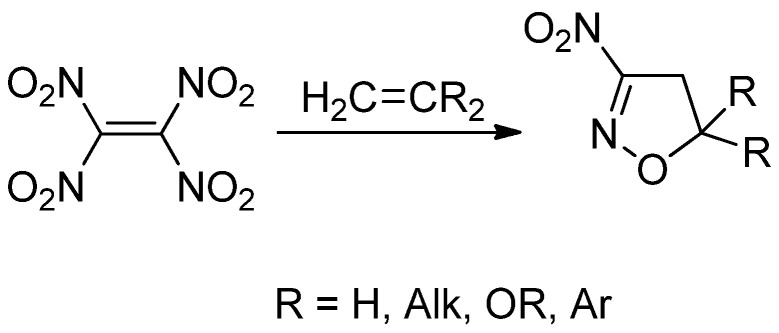
Conversion of tetranitroethene into 3-nitroisoxazoline derivatives.

On the other hand, some 5,5-gem-substituted 3-nitro-2-isoxazolines are formed as a result of the reaction between tetranitroethene and geminal substituted ethenes. The drawback of this strategy is the preparation of the starting tetranitroethene via the decomposition of unstable hexanitroethane [40,41] (Scheme 5).

**Scheme 6:**
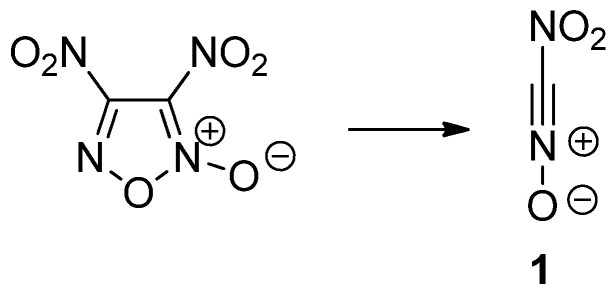
Decomposition of dinitrofuroxane leading to formonitrile N-oxide **1**.

All of the aforementioned strategies are not universally applicable and cannot be treated as general procedures for the preparation of a wide range of 3-nitro-2-isoxazilines. The [3+2] cycloaddition reaction involving formonitrile N-oxide **1**, which can be obtained via the thermal decomposition of dinitrofuroxan [42] (Scheme 6), can be considered a potential universal protocol for the preparation of these compounds.

Unfortunately, these reactions have not yet been the subject of deeper systematic studies. In the literature, one may only find short communications regarding the 32CA reaction of selected COOMe-substituted alkenes [43]. This study does not, however, conduct a systematic exploration of the main problem. It should be noted that at this point the synthesis and properties of other, simply substituted nitrile N-oxides are understood substantially better (F [44,45], Cl [46,47,48], Br [49,50,51], and CN [52,53,54]).

Some similar reactions involving nitrile N-oxide **1** were tested under catalytic conditions with the presence of an RTIL (Room-Temperature Ionic Liquid). It should, however, be noted that the latest discoveries in the field of RTIL-catalyzed cycloaddition reactions indicate that its selectivity and molecular mechanism is completely different from analogous thermal reactions [55,56,57]. So, the main problem requires a deeper and systematic exploration. Due to the aforementioned issues, in the present work, we carried out a comprehensive DFT computational study regarding the 32CA processes involving formonitrile N-oxide **1** and a series of electron-rich mono- and gem-substituted alkenes.

**Scheme 7:**
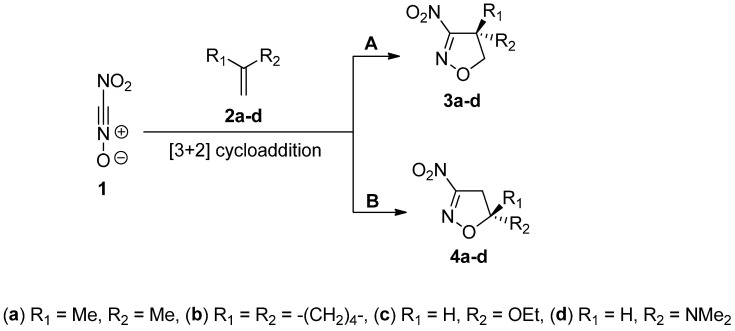
Theoretically possible paths of the cycloaddition of nitro-substituted formonitrile N-oxide **1** with electron-rich alkenes **2a**–**d**.

Due to the expected electrophilic nature of the nitro-substituted formonitrile N-oxide **1** determined by the presence of a strong EWG (*Electron Withdrawing Group*) character of the nitro group, we selected nucleophilic-activated alkenes, such as isobutene **2a**, methylenecyclopentane **2b**, ethyl-vinyl ether **2c**, and N,N-dimethyl-vinyl amine **2d,** as model alkenes for carrying out the main cycloadditions. These alkenes were recently tested by us as components of different types of cycloadditions involving electrophilic agents [58,59,60] (Scheme 7).

Independently of the energetic aspects and their influence on the regioselectivity, the main processes also require a detailed mechanistic evaluation. According to the actual state of knowledge, different types of molecular mechanisms can occur during the [3+2] cycloaddition reactions: (a) polar mechanisms (one-step synchronous mechanism, one-step–two-stage asynchronous mechanism, and stepwise zwitterionic mechanism) [61,62,63,64], or (b) non-polar mechanisms (one-step synchronous mechanism, one-step–two-stage asynchronous mechanism, and stepwise biradical mechanism) [65,66].

Thus, in this work, we aimed to (i) analyze the of the global and power local interactions in the light of the Conceptual Density Functional Theory (CDFT) for predicting the nature of cycloaddition and regioselectivity; (ii) consider thermodynamic factors; and (iii) perform a full exploration of the reaction profiles for understanding the molecular mechanism as well as kinetic aspects of the main processes. The aforementioned analyses were performed according to DFT quantum chemical calculations.

## 2. Results and Discussion

### 2.1. Power of the Global and Local Interactions in the Context of the CDFT 

Analysis of the electronic properties of organic molecules is an important and universal tool for predicting their reactivity [67,68,69]. This approach was recently and successfully applied to interpret the reactivity of different types of molecular segments and the course of many bimolecular organic processes [70,71,72,73,74].

**Scheme 8:**
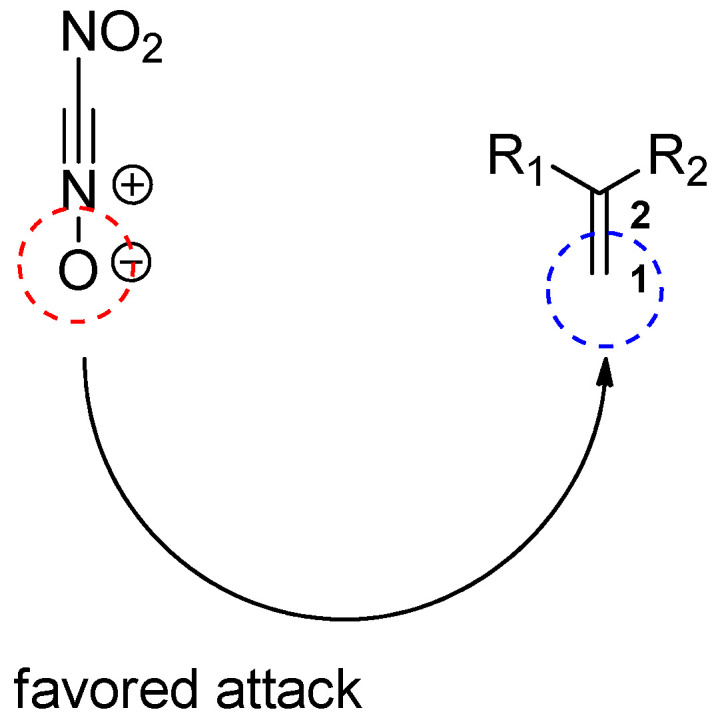
Most efficient local electronic interactions of nitro-substituted formonitrile N-oxide **1** with electron-rich alkenes **2a**–**d**.

Global and local electronic properties of the main compounds were estimated according to equations recommended in the literature (see Computational Details) and presented in Table 1. The analysis of the values of electronic chemical potential clearly shows that, in the case of all considered reaction pairs, the electron density transfers from the alkene molecule to nitrile N-oxide. Thus, the considered processes—according to the *Domingo* convention [75]—should be classified as Forward Electron Density Flux (FEDF) processes. The global electrophilicity of nitrile N-oxide **1** is equal to 3.68 eV. Therefore, it should be classified as a strong electrophile [76]. In contrast, the global nucleophilicity index of **1** is less than 1 eV. Thus, the potential nucleophilic properties of this compound is evidently marginal. On the other hand, all four alkenes, **2a**–**d**, are characterized by a global electrophilicity index equal to or less than 0.6 eV. Hence, their electrophilic properties are marginal. In contrast, all these compounds exhibit greater nucleophilic properties as their respective global nucleophilicities are equal to or more than 2.5 eV. The most nucleophilic agent within the considered group is N,N-dimethylvinylamine **2d**, whereas the weakest nucleophilic agent is isobutene **2a** (Scheme 8). 

The analysis of the electronic properties allow for the prediction of the potential regioselectivity of bimolecular processes. This is possible because these types of reactions occur under the control of local interactions between the most electrophilic reaction center on the first molecule and the most nucleophilic center at on second one [72,77]. It was found that the more electrophilic center within the CNO moiety of N-oxide **1** is localized on the oxygen atom (ω_O_ = 0.34 eV). On the other hand, the more nucleophilic center at the >C=C< moiety of the alkene is always localized on the terminal carbon atom (N_1_ = 1.68–2.23 eV). The interaction of these centers stimulates the energetical preference for the formation of 3-nitro-4,4-substituted 2-isoxazolines **3a**–**d**.

### 2.2. Thermodynamic Considerations

Within the framework of the thermodynamic analysis of the main cycloadditions, we treated all competing reactions as independent processes. To perform this analysis, the values of the thermodynamic functions of the substrates and products were required. Unfortunately, for the compounds that are the subject of our study, these data are not available in the chemical literature. Therefore, we used data from DFT quantum chemical calculations to determine them. Based on estimated enthalpies and entropies of formation of substrates and products, we calculated the enthalpy and entropy values for each cycloaddition reaction, followed by the *Gibbs* free energies of the reaction. The results of thermodynamic calculations for the considered reaction paths are presented in Table 2.

The data obtained via DFT calculations show (Table 2) that, independently of the alkene structures, in the toluene solution, the Gibbs free energies of the reaction are strongly negative for both considered cycloaddition paths. During this period, the reaction channels leading to less sterically crowded 5,5-disubstituted 2-isoxazolines (path **B**) are always preferred. The replacement of toluene with the more polar solvent (nitromethane) did not change this tendency. Thus, the thermodynamic preference for the formation of 3-nitro-5,5-di(R)-2-isoxazolines can be considered as a general rule in the context of the main reactions.

### 2.3. Exploration of Reaction Profiles

Firstly, we analyzed the energy reaction profiles for the 32CA of nitro-substituted formonitrile N-oxide **1** and isobutene **2a**. It was found that the nature of the reaction trajectories are qualitatively similar for both regioisomeric cycloaddition channels considered. In particular, in the framework of both considered profiles, two critical points (pre-reaction molecular complex, MC, and the transition state, TS) between the valleys of the starting molecules and products were detected. The quantitative description of the aforementioned transformations is, however, substantially different.

**Figure 1:**
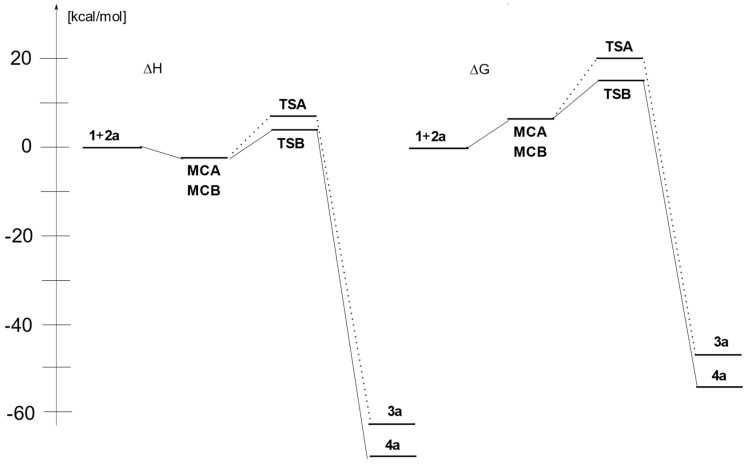
Enthalpy and *Gibbs* free energy profiles the [3+2] cycloaddition reaction between the nitro-substituted formonitrile N-oxide **1** and isobutene **2a** in the toluene solution according to the wb97xd/6-311+G(d) (PCM) calculations.

In the initial phase of the reaction progress, respective pre-reaction molecular complexes are formed in the case of both 32CA pathways (**MCA** and **MCB** for paths **A** and **B**, respectively). The reduction in enthalpy of the molecular system by about 2.3–2.5 kcal/mol is a result of this transformation (Table 2, Figure 1). At the same time, however, the entropy of the molecular system is substantially reduced. As a result, the Gibbs free energies of formation for both MCs are positive. This excludes the possibility of the existence of **MCA** and **MCB** complexes as thermodynamically stable intermediates. Within the MC, substructures of addends adopt orientations that stimulate maximum stable coulombic interactions. Any new sigma bond, however, are not created at this stage. The key interatomic distances are beyond the range typical for C-C and O-C bonds formed in the transition state (Table 3, Figure 1). Additionally, the electron density transfer between substructures is not observed. Thus, the detected structures cannot be considered as charge–transfer complexes [78,79]. Similar intermediates were recently detected in many 32CAs involving different types of TACs [80,81].

The gradual reduction in interatomic distances between the nitrile N-oxide **1** and isobutene **2a** substructures along the reaction coordinate leads directly to the transition state (**TSA** and **TSB** for paths **A** and **B,** respectively). This stage is associated with an increase in enthalpy of the reaction system by 7.7 kcal/mol in the case of path **A** and 3.6 kcal/mol in the case of path **B** (Table 2, Figure 1). Including entropic factors in the energetic considerations yield Gibbs free energies of activation equal to 19.9 kcal/mol and 13.6 kcal/mol, respectively, for paths **A** and **B**. Thus, in the considered system of competing reactions, the formation process of isoxazoline **3a** should be considered as forbidden from a kinetic point of view. Therefore, the predicted regioselectivity of the cycloaddition is fully different from that suggested by earlier analysis of local electronic interactions. The regiodirection of the considered reaction is predominantly determined by steric repulsion involving the germinal methyl groups derived from the isobutene molecule. In contrast, analogous 32CAs involving electron-rich nitrile N-oxides and electrophilic alkenes are fully determined by the nature of global and local electronic interactions [23,24,70]. 

**Figure 2:**
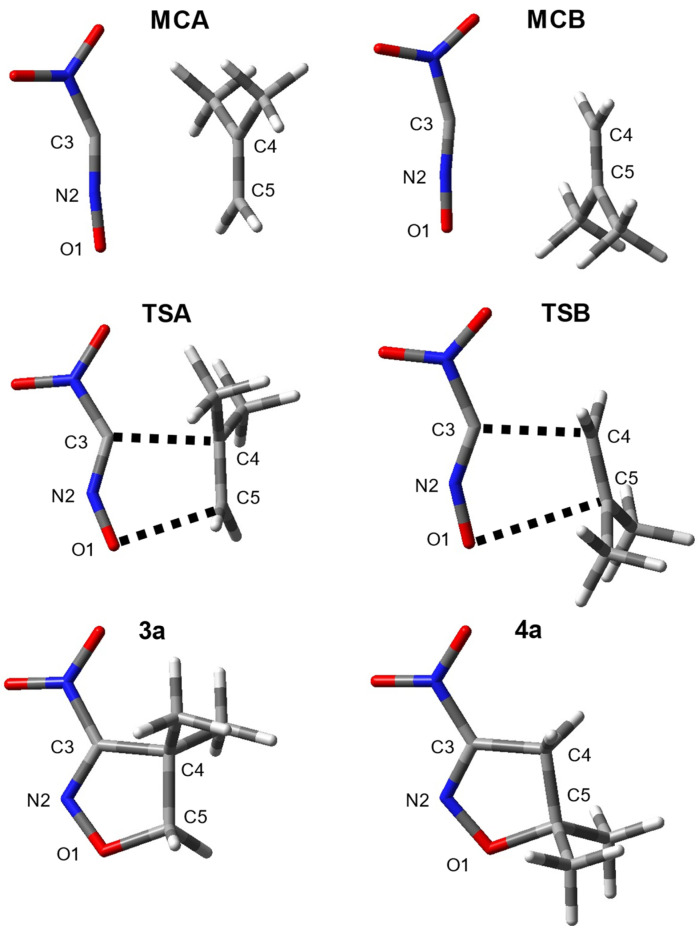
Views of critical structures of the [3+2] cycloaddition reaction between the nitro-substituted formonitrile N-oxide **1** and isobutene **2a** in the toluene solution according to the wb97xd/6-311+G(d) (PCM) calculations.

**Figure 3:**
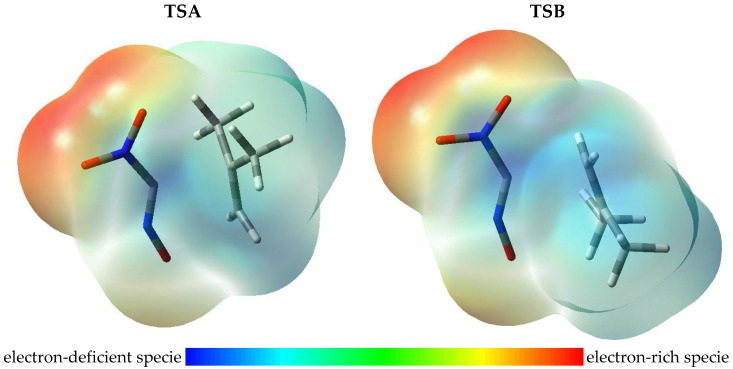
Molecular electrostatic potential maps for **TS4** and **TS5** structures of the [3+2] cycloaddition reaction between the nitro-substituted formonitrile N-oxide **1** and isobutene **2a**. Negative charges are colored in red, while negligible charges are colored in green.

Within both optimized TSs, two new sigma-bonds are formed. The synchronicity of this process is, however, substantially different in the case of **TSA** than in the case of **TSB** (Table 2, Figure 2). In particular, in **TSA**, key interatomic distances C3-C4 and C5-O1 are equal to 2.360Å and 2.434Å, respectively. However, within **TSB,** respective interatomic distances are equal to 2.292Å and 2.865Å, respectively. The asynchronicity is, however, not sufficient to enforce a stepwise, zwitterionic mechanism [82,83,84,85]. The formation of new bonds is accompanied by the redistribution of electron density. In particular, the electron density is transferred gradually from the alkene substructure to the nitrile oxide substructure (GEDT = 0.16–0.16 e). This confirms that the currently analyzed reaction should be classified as a Forward Electron Density Flux (FEDF) process. Molecular electrostatic potential maps for **TS4** and **TS5** structures are presented in Figure 3. This analysis evidently confirms the polar nature of localized TSs. Further conversion of the TS structures leads directly to the following respective products—**3a** in the case of pathway **A** and **4a** in the case of pathway **B**. 

Replacement of isobutene **3a** in the 32CA environment with the more nucleophilic alkenes, such as **3b**, **3c** and **3d,** does not change the nature of the energy profiles for all considered cycloaddition channels. The description of respective profiles as well as optimized critical points are, however, changed. In particular, the energy barrier on the kinetically favored cycloaddition pathway **B** is clearly reduced with the increase in the global nucleophilicity of the alkene. At the same time, the polarity and asynchronicity of respective transition structures are more evident. However, we cannot detect theoretically possible zwitterionic intermediates. A similar effect is also determined due to the influence of the polarity solvent on the reaction progress (Table 2, Table 3 and Table 4). Thus, the described scheme and mechanism should be treated as a general rule for a wide range 32CAs involving nitro-substituted formonitrile N-oxide **1.**

## 3. Computational Details 

The DFT calculations were performed using the wb97xd functional and 6-311+G(d) basis sets and the software implemented within the Gaussian package [86]. The PlGrid infrastructure (“Ares” cluster) in the computing center “Cyfronet” was applied. A similar computational level has already been successfully used to explore the mechanistic aspects of different types of cycloaddition processes, including the [3+2] cycloaddition reactions involving allenyl-type TACs [87,88,89] as well as different-type transformations of hydrocarbons [90]. All localized stationary points have been characterized using vibrational analysis. It was found that the starting molecules, intermediates, and products had positive eigenvalues in their Hessian matrices, whereas all transition states (**TS**) showed only one negative eigenvalue in their Hessian matrices. Intrinsic reaction coordinate (IRC) calculations have been performed for all localized transition states. The presence of the solvent in the reaction environment (toluene and nitromethane) has been included using the IEFPCM algorithm [91]. The Global Electron Density Transfer (GEDT) [92] was calculated according to Formula (1):

GEDT = −ΣqA(1)

where qA is the net charge, and the sum is taken including all the atoms of nitroalkene.

Global electronic properties of the reactants were estimated according to the equations recommended by *Parr* and *Domingo* [64,66,67,93,94,95,96]. According to *Domingo*’s recommendation, for this purpose, the B3LYP/6-31G(d) level of theory was used. In particular, the electronic chemical potentials (μ) and chemical hardness (η) were evaluated in terms of one-electron energies of FMO (E_HOMO_ and E_LUMO_) using Equations (2) and (3):

μ ≈ (E_HOMO_ + E_LUMO_)/2 (2)

η ≈ E_LUMO_ − E_HOMO_ (3)

Next, the values of μ and η were used for the calculation of global electrophilicity (ω) according to Formula (4):

ω = μ^2^/2η (4)

Subsequently, global nucleophilicity (N) [66] could be expressed in terms of Equation (5):

N = E_HOMO_ − E_HOMO (tetracyanoethene)_ (5)

The local electrophilicity (ω_k_) [67] condensed to atom *k* was calculated by projecting the index ω onto any reaction center *k* in the molecule by using the *Parr* function P^+^_k_ (6):

ω_k_ = P^+^_k_·ω (6)

The local nucleophilicity (N_k_) [67] condensed to atom *k* was calculated using global nucleophilicity N and *Parr* function P^−^_k_ according to Formula (7):

N_k_ = P^−^_k_·N (7)

## 4. Conclusions

Our comprehensive DFT study regarding [3+2] cycloaddition involving nitro-substituted formonitrile N-oxide **1** and electron-rich alkenes shed light on the reaction regioselectivity and molecular mechanism. In particular, the wb97xd/6-311+G(d) (PCM) calculations show that independently of the alkene structure as well as of the polarity of the solvent, the thermodynamic factors allow for the formation of stable cycloadducts within considered cycloadditions. Next, the analysis of the local electronic properties suggest the preference for the formation of 4-R-isoxazolines. In contrast, kinetic factors obtained via the full exploration of energy profiles lead to a preference for the formation of 5-substituted 2-isoxazolines. This clearly demonstrates the influence of steric factors on the course of the reaction. The respective transition states leading to the aforementioned products are always asynchronous, but all attempts to locate the hypothetical zwitterionic intermediates along the cycloaddition paths were unsuccessful. In summary, it should be underlined that the scheme and mechanism described in this study should be treated as a general occurrence for a wide range of 32CA involving nitro-substituted formonitrile N-oxide.

## Figures and Tables

**Table 1:** Global (electronic chemical potentials μ, chemical hardness η, global electrophilicities ω, and global nucleophilicities N) and local (local Parr spin density functions P^+^_k_ and P**^−^**_k_, local nucleophilicities N_k_, and local electrophilicities ω_k_) electronic properties of nitro-substituted formonitrile N-oxide **1** and electron-rich alkenes **2a**–**d**.

	Global Properties				Local Properties 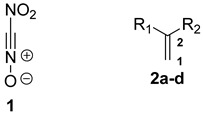							
	μ [eV]	η [eV]	ω [eV]	N [eV]	P^−^_2_	P^−^_1_	N_2_ [eV]	N_1_ [eV]	P^+^_C_	P^+^_O_	ω_C_ [eV]	ω_O_ [eV]
**1**	−5.98	4.85	3.68	0.72					0.048	0.091	0.18	0.34
**2a**	−2.83	7.37	0.55	2.60	0.295	0.646	0.77	1.68				
**2b**	−2.87	6.95	0.59	2.77	0.266	0.636	0.74	1.76				
**2c**	−2.39	6.97	0.41	3.25	0.057	0.577	0.18	1.87				
**2d**	−1.87	6.50	0.27	3.99	0.110	0.557	0.44	2.23				

**Table 2:** Key thermodynamic and kinetic parameters for 32CA of nitro-substituted formonitrile N-oxide **1** with electron-rich alkenes **2a**–**d** according to the wb97xd/6-311+G(d) (PCM) calculations (ΔH and ΔG are in kcal/mol; ΔS are in cal/molK).

Reaction	Transition	Toluene			Nitromethane		
		ΔH	ΔG	ΔS	ΔH	ΔG	ΔS
**1+2a**	**1+2a→MCA**	−2.3	5.9	−27.4	−1.8	6.3	−27.4
	**1+2a→TSA**	7.7	19.6	−39.8	8.5	20.4	−39.9
	**1+2a→3a**	−63.5	−47.9	−52.2	−64.7	−49.2	−51.9
	**1+2a→MCB**	−2.5	6.6	−30.4	−3.4	5.7	−30.7
	**1+2a→TSB**	3.6	13.6	−33.3	4.1	13.8	−32.7
	**1+2a→4a**	−70.0	−54.6	−51.7	−71.4	−55.7	−52.6
**1+2b**	**1+2b→MCA**	−4.4	4.3	−29.1	−3.3	4.9	−27.3
	**1+2b→TSA**	8.4	20.5	−40.9	9.1	21.6	−41.8
	**1+2b→3b**	−64.3	−49.6	−49.3	−65.4	−50.7	−49.2
	**1+2b→MCB**	−3.9	4.7	−28.7	−2.9	5.4	−27.9
	**1+2b→TSB**	2.0	12.6	−35.6	2.3	13.0	−35.8
	**1+2b→4b**	−69.2	−56.6	−42.1	−70.4	−58.2	−41.0
**1+2c**	**1+2c→MCA**	−7.0	4.6	−38.9	−5.5	5.5	−37.0
	**1+2c→TSA**	5.7	18.2	−41.8	6.5	18.9	−41.4
	**1+2c→3c**	−59.9	−45.1	−49.4	−60.6	−45.7	−49.8
	**1+2c→MCB**	−5.0	3.7	−29.2	−1.9	3.8	−19.1
	**1+2c→TSB**	0.5	11.5	−37.1	1.4	12.0	−35.6
	**1+2c→4c**	−70.5	−55.9	−49.1	−71.0	−56.4	−48.9
**1+2d**	**1+2d→MCA**	−4.3	3.1	−24.8	−3.5	4.1	−25.3
	**1+2d→TSA**	5.1	15.9	−36.0	5.7	16.5	−36.2
	**1+2d→3d**	−58.5	−44.8	−46.2	−58.9	−44.8	−47.2
	**1+2d→MCB**	−4.6	3.1	−25.8	−3.7	4.2	−26.5
	**1+2d→TSB**	−4.0	6.6	−35.7	−3.6	6.8	−35.2
	**1+2d→4d**	−67.4	−53.5	−46.5	−68.3	−54.3	−46.8

**Table 3:** Key parameters of critical structures of the 32CA of nitro-substituted formonitrile N-oxide **1** with electron-rich alkenes **2a**–**d** in the toluene solution according to the wb97xd/6-311+G(d) (PCM) calculations (ΔH and ΔG are in kcal/mol; ΔS are in cal/molK).

Reaction	Structure	Interatomic Distances [Å]					GEDT [e]
		O1–N2	N2–C3	C3–C4	C4–C5	C5–O1	
**1+2a**	**1**	1.181	1.162				
	**2a**				1.332		
	**MCA**	1.179	1.163	3.299	1.332	3.344	0.00
	**TSA**	1.195	1.215	2.360	1.364	2.434	0.16
	**3a**	1.347	1.271	1.510	1.546	1.455	
	**MCB**	1.184	1.157	3.216	1.333	3.416	0.00
	**TSB**	1.196	1.199	2.292	1.357	2.865	0.13
	**4a**	1.343	1.271	1.487	1.542	1.489	
**1+2b**	**2b**				1.329		
	**MCA**	1.179	1.167	3.596	1.330	3.565	0.00
	**TSA**	1.195	1.213	2.342	1.361	2.461	0.20
	**3b**	1.356	1.272	1.503	1.534	1.450	
	**MCB**	1.179	1.167	3.519	1.330	3.556	0.00
	**TSB**	1.199	1.198	2.268	1.354	3.037	0.14
	**4b**	1.344	1.271	1.488	1.532	1.489	
**1+2c**	**2c**				1.329		
	**MCA**	1.179	1.168	3.854	1.331	5.153	0.00
	**TSA**	1.201	1.217	2.264	1.365	2.484	0.12
	**3c**	1.349	1.272	1.510	1.531	1.453	
	**MCB**	1.179	1.168	3.739	1.328	3.475	0.00
	**TSB**	1.200	1.192	2.357	1.351	3.045	0.13
	**4c**	1.360	1.269	1.488	1.524	1.467	
**1+2d**	**2d**				1.341		
	**MCA**	1.187	1.164	3.271	1.347	4.557	0.00
	**TSA**	1.204	1.227	2.680	1.376	2.227	0.22
	**3a**	1.351	1.272	1.520	1.533	1.453	
	**MCB**	1.192	1.164	2.890	1.349	4.070	0.00
	**TSB**	1.206	1.186	2.370	1.365	3.643	0.13
	**4d**	1.346	1.272	1.487	1.533	1.515	

**Table 4:** Key parameters of critical structures of the 32CA of nitro-substituted formonitrile N-oxide **1** with electron-rich alkenes **2a**–**d** in the nitromethane solution according to the wb97xd/6-311+G(d) (PCM) calculations (ΔH and ΔG are in kcal/mol; ΔS are in cal/molK).

Reaction	Structure	Interatomic Distances [Å]					GEDT [e]
		O1–N2	N2–C3	C3–C4	C4–C5	C5–O1	
**1+2a**	**1**	1.183	1.158				
	**2a**				1.333		
	**MCA**	1.180	1.161	3.308	1.333	3.334	0.00
	**TSA**	1.195	1.214	2.369	1.364	2.458	0.18
	**3a**	1.346	1.271	1.509	1.547	1.459	
	**MCB**	1.181	1.164	3.416	1.333	4.015	0.00
	**TSB**	1.198	1.197	2.283	1.357	2.995	0.15
	**4a**	1.342	1.272	1.485	1.543	1.495	
**1+2b**	**2b**				1.330		
	**MCA**	1.181	1.164	3.586	1.331	3.602	0.00
	**TSA**	1.196	1.212	2.352	1.360	2.484	0.21
	**3b**	1.354	1.272	1.502	1.535	1.455	
	**MCB**	1.181	1.164	3.529	1.331	3.711	0.00
	**TSB**	1.201	1.196	2.271	1.353	3.166	0.16
	**4b**	1.341	1.272	1.486	1.534	1.495	
**1+2c**	**2c**				1.330		
	**MCA**	1.180	1.166	3.813	1.332	4.933	0.00
	**TSA**	1.200	1.216	2.283	1.364	2.513	0.13
	**3c**	1.346	1.273	1.510	1.531	1.457	
	**MCB**	1.182	1.161	3.417	1.330	3.381	0.00
	**TSB**	1.200	1.190	2.361	1.351	3.146	0.15
	**4c**	1.357	1.270	1.487	1.525	1.475	
**1+2d**	**2d**				1.343		
	**MCA**	1.186	1.153	3.290	1.348	4.536	0.00
	**TSA**	1.202	1.226	2.756	1.375	2.257	0.25
	**3a**	1.349	1.273	1.521	1.532	1.457	
	**MCB**	1.191	1.164	2.892	1.350	4.082	0.00
	**TSB**	1.205	1.179	2.443	1.363	3.737	0.23
	**4d**	1.343	1.273	1.486	1.533	1.526	

## Data Availability

The raw data supporting the conclusions of this article will be made available by the authors on request.
